# Ocular surface squamous neoplasia in Northern Thailand: a 16-year review

**DOI:** 10.1186/s12886-022-02340-y

**Published:** 2022-03-12

**Authors:** Napaporn Tananuvat, Muanploy Niparugs, Damrong Wiwatwongwana, Nirush Lertprasertsuk, Pongsak Mahanupap

**Affiliations:** 1 Department of Ophthalmology, Faculty of Medicine, Chiang Mai University, 110 Intavaroros Road, Chiang Mai, 50200 Thailand; 2 Department of Pathology, Faculty of Medicine, Chiang Mai University, 110 Intavaroros Road, Chiang Mai, 50200 Thailand

**Keywords:** Cornea, Conjunctiva, Intra-epithelial neoplasia, Ocular surface squamous neoplasia, Squamous cell carcinoma, Thailand

## Abstract

**Purpose:**

To evaluate clinical characteristics, treatments, and outcomes in patients with ocular surface squamous neoplasia (OSSN) at a tertiary center in Northern Thailand.

**Methods:**

Patients diagnosed with either corneal-conjunctival intraepithelial neoplasia (CIN) or squamous cell carcinoma (SCC) from May 2000 to December 2015, were recruited. The patients’ demographics, symptoms, clinical characteristics, cytopathology, treatments, and outcomes were reviewed.

**Results:**

Overall 171 eyes from 168 patients, 92 eyes were CIN and 79 eyes were SCC. Males were affected in 65.5%. The mean age was 58.8 ± 16.8 (29–99) years. In most cases (60.3%), the tumors were located at the limbus. The most common clinical characteristic was papilliform appearance (46.2%). Human immunodeficiency virus (HIV) infection was found in 37 (22.0%) patients with a mean age of 40.5 ± 7.7 years. The treatments and outcomes were evaluated in 136 eyes whose main initial treatment was wide excision with adjunctive cryotherapy (47.8%), followed by topical mitomycin C (30.9%). The mean follow-up time after treatment was 20.8 ± 2.2 (3–110) months and the recurrence occurred in 18 eyes (13.2%) during the follow-up period. The mean recurrence-free time (months) for CIN was significantly longer than that of SCC (81.3 ± 10.0 [95%CI 61.5 – 101.1] vs 33.2 ± 4.6 [95%CI 24.0 – 42.3], *p* = 0.030). SCC was the only significant risk factor that influences the recurrence of the tumors with the adjusted hazard ratio of 5.69 (*p* = 0.005).

**Conclusion:**

OSSN in Northern Thailand usually involved a limbal area and presented as a papilliform mass. HIV infection should be suspected in young patients. CIN had better outcomes after treatments than invasive SCC.

## Introduction

Ocular surface squamous neoplasia (OSSN) is the most frequent acquired non-melanocytic tumor of the ocular surface [1]. It denotes neoplastic lesions of squamous epithelial origin on the cornea and conjunctiva and covers a spectrum of diseases ranging from pre-invasive corneal-conjunctival intraepithelial dysplasia (CIN) to invasive squamous cell carcinoma (SCC) which the dysplastic epithelial cells have penetrated the basement membrane [2].

The incidence for OSSN ranges varies regionally between 0.03–3.5 cases/year/100,000 [3, 4]. The incidence in Africa was reported about 9–10 times higher than in Europe and North America [3]. Males, Caucasians, and residents of lower latitudes (closer than 30 latitudes to the equator) are at the highest risk of OSSN [2, 4, 5]. The etiology of OSSN is multifactorial with an interplay of several factors including advanced age, male gender, exposure to solar ultraviolet radiation, race, human papillomavirus (HPV), immunosuppression, and human immunodeficiency virus (HIV) infection [2–9].

Although OSSN is considered a low-grade malignancy, early detection with an appropriate and prompt treatment is essential in decreasing the risk of metastasis and recurrence after treatments. Furthermore, the proper treatments minimized treatment-related morbidities and improved disease control with good functional visual outcomes. Currently, there is a wide range of surgical and non-surgical treatment options for the management of OSSN; however, there has been no consensus on disease management [7]. Previously, surgical excision was the mainstay of primary treatment for OSSN, nevertheless, the recurrence rates after surgery have been reported to be as high as 15–56% [2, 10, 11]. A wide surgical excision with adjunctive cryotherapy results in lower recurrence rates compared to the surgical excision only [10]. Because only surgical excision may not completely eradicate the microscopic tumor cells at or beyond the edge of the surgical margin. Over the past 20 years, several alternative therapies have been proposed, including topical treatment with mitomycin C (MMC), 5-fluorouracil (5-FU), and interferon-α2b (IFN) [12–16]. Advantages of topical chemotherapy include avoidance of surgical complications and the ability to treat microscopic or subclinical disease at a different site from the clinically evident tumor [13, 15]. Currently, topical chemotherapy is a preferred treatment, either as a primary monotherapy or in combination with surgical excision as a preoperative chemo-reduction treatment or a postoperative adjunct treatment to prevent tumor recurrence [12, 16].

Since there has been limited study regarding the OSSN in Thailand which is a tropical country located close to the equator. This study aimed to evaluate the clinical characteristics, treatments, and treatment outcomes in patients with OSSN in Northern Thailand.

## Materials and methods

This retrospective cohort study included all patients who were diagnosed with either corneal and/or conjunctival intraepithelial neoplasia (CIN) or squamous cell carcinoma (SCC) at Chiang Mai University Hospital, a tertiary eye center in Northern Thailand, over 16 years from May 2000 to December 2015. The protocol of this study was approved by the Research and Ethics Committee, Faculty of Medicine, Chiang Mai University (Study code: OPT-2558–02,788) and complied with the tenets of the Declaration of Helsinki.

Medical records of all patients diagnosed as CIN or SCC were reviewed. The patients’ demographics including age, gender, laterality, other ocular diseases and underlying systemic diseases, and the serology for HIV infection were collected. Clinical characteristics, which were symptoms duration, presenting clinical features and tumor characteristics, as well as other associated findings, were analyzed. The diagnosis of CIN or SCC in most cases was made according to the tissue histopathologic findings. However, in some patients who did not undergo incisional biopsy or surgical excision, the diagnosis was made by impression cytology (IC). The technique of IC was described in our previous study [17]. In brief, a 5-mm strip of cellulose acetate filter paper was applied with gentle pressure on the ocular surface lesion after the instillation of topical anesthesia. The paper was immediately transferred into a fixative solution and then transfer to a cytologist who was an expert in ocular cytology (NL) for analysis.

### Treatment regimen and outcomes

Treatment options were carefully considered for each individual patients’ conditions including the cytopathologic findings, the extension of the tumor, the patient’s general condition whether he/she was suitable for surgery or not, as well as the patient’s compliance to each treatment option. Surgical excision was indicated in patients who had a small lesion and required histopathologic section or patients who could not comply with topical treatment. The main surgical technique was wide excision with adjunctive cryotherapy. In short, the lesions were excised with at least 3–4 mm of clinically uninvolved conjunctival margin combined with using a double rapid freeze–thaw technique cryotherapy at the conjunctival edges and involved limbal zone. For tumor with scleral extension, lamellar sclerectomy was done. If there was no scleral involvement, the bared scleral bed was avoided as much as possible for excessive cryotherapy to prevent postoperative hypotony. If the area of surgical resection was large, an amniotic membrane graft was applied to cover the resection site. In the case of corneal intraepithelial neoplasia, superficial keratectomy was done, followed by the application of 95% alcohol.

Besides the surgical treatment, topical chemotherapy was selected as a monotherapy inpatient with a diffuse lesion or who could not undergo surgery. Also, topical chemotherapy was used as a chemo-reduction before wide excision in cases with large lesions to prevent complications related to the surgery. The regimen was a two-week cycle of a topical MMC 0.02% application four times daily during a one-week-on period followed by no application during a one-week-off period. The patients were instructed to close their eyes for five minutes after the administration of topical MMC. Topical lubricants were prescribed to reduce drug toxicity. The extension of cycle treatment was considered according to either the clinical response of the lesion or the development of complications.

The treatment outcomes were categorized into completely resolved; partially resolved; not improved; and recurrence. The recurrence of disease was defined as a recurrent mass in the same eye after complete resolution of the tumor from the primary previous treatment. Patients with a follow-up time less than three months after treatment were excluded for analysis of the treatment outcomes.

### Statistical analysis

All proportions were presented as numbers and percentages. Continuous data were presented as mean and standard deviation (SD) in normally distributed data or median and interquartile range in non-normally distributed data. Kaplan–Meier estimator and group compared with the log-rank test were used to calculate the recurrence-free time and compare the median survival time between SCC and CIN patients. Cox regression with stepwise analysis was used to evaluate factors influencing the recurrence. A *p*-value < 0.05 was considered statistically significant. Data were analyzed by using SPSS program version 22.0 (SPSS Inc., Chicago, IL, USA).

## Results

### Patients’ demographics data and presenting symptoms

A total of 168 OSSN patients were reviewed. There were 110 (65.5%) males and 58 (34.5%) females, with a mean ± SD age of 58.8 ± 16.8 years (range 29–99). The tumors involved the right eye in 73 patients (43.5%), the left eye in 92 patients (54.7%), and bilateral eyes in three patients (1.8%). The HIV serology was assessed in 80 patients (47.6%) and the HIV seropositive was found in 37 patients (22.0%) who had a mean ± SD age of 40.5 ± 7.7 (range 29–57) years.

The main presenting symptoms were a lump on the eye, followed by eye irritation or pain, and visual impairment. (Table 1) The mean ± SD duration of the symptoms was 7.0 ± 10.0 (range 1- 60) months. The lesion was accidentally found during eye check-ups or eye examination for other eye problems in 5.2% of patients.

**Table 1:** Patients’ demography and presenting symptoms

Patients’ demographics (*N* = 168 patients)	
Age (year)	
- Mean ± SD	58.8 ± 16.8
- Range	29–99
Gender (N, %)	
- Male	110 (65.5%)
- Female	58 (34.5%)
Presenting symptoms (N, %)	
- Mass	98 (58.3%)
- Eye irritation or pain	35 (20.8%)
- Visual impairment	26 (15.5%)
- None	9 (5.4%)
Laterality (N, %)	
- Right eye	73 (43.5%)
- Left eye	92 (54.7%)
- Bilateral eye	3 (1.8%)
Serology for HIV (N, %)	
- Positive	37 (22.0%)
- Negative	43 (25.6%)
- N/A	88 (52.4%)

### Tumor characteristics and pathological study

**Fig. 1:**
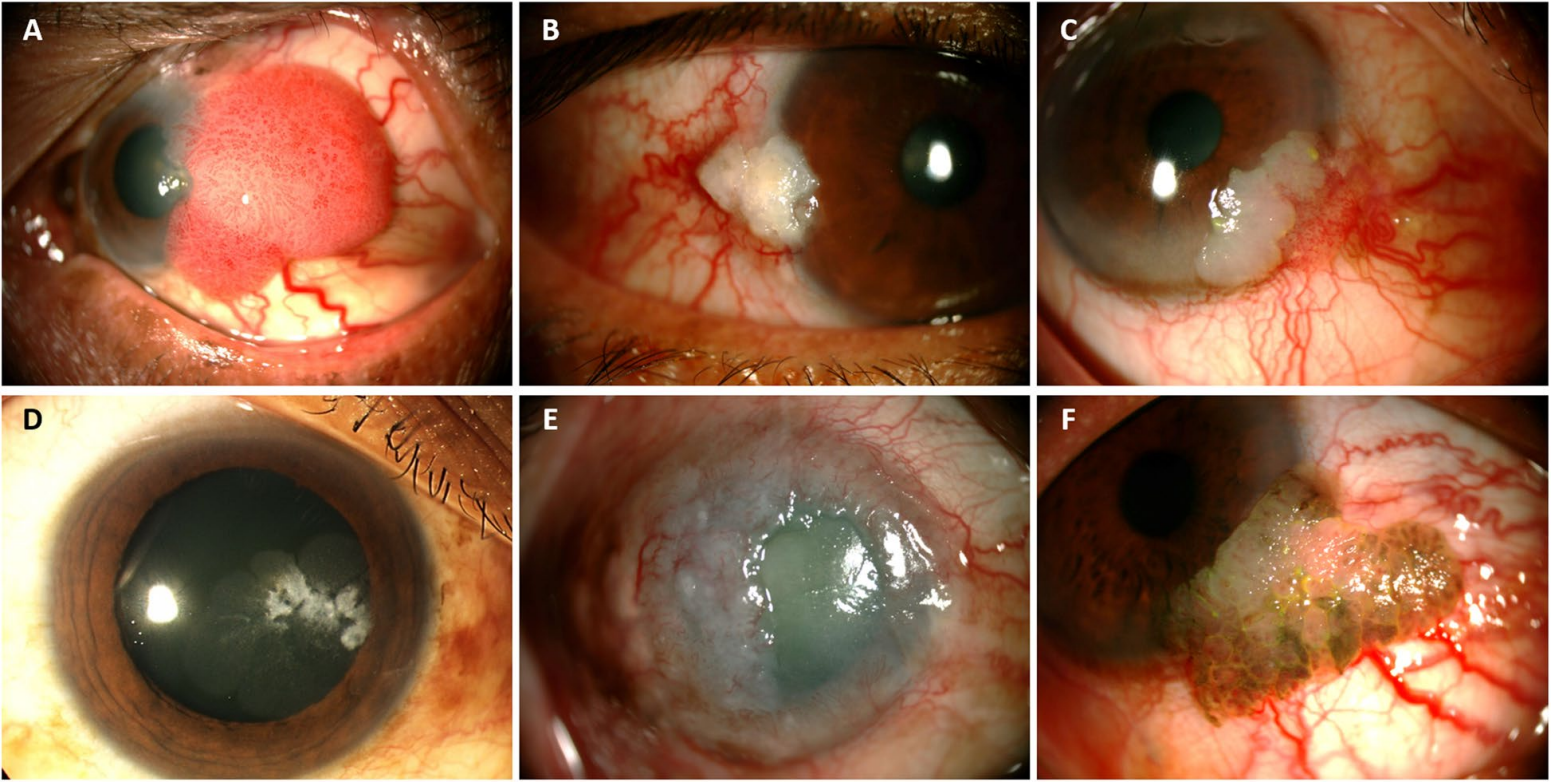
Presenting Clinical Characteristics of the Ocular Surface Squamous Neoplasia: **A** Papilliform mass, **B** Leukoplakia lesion, **C** Gelatinous mass, **D** Combined form, **E** Diffuse/ulcerated mass, and **F** Pigment-associated mass

Among 171 eyes of 168 patients, 92 eyes (53.8%) were CIN and 79 eyes (46.2%) were SCC. Five eyes (2.9%) were the recurrent disease at presentation with a history of previous treatment elsewhere. The diagnosis was made according to tissue histopathologic study from either incisional and/or excisional biopsy in 85 eyes (49.7%), impression cytology in 47 eyes (27.5%), and both in 39 eyes (22.8%). Almost half of the CIN (43/92) cases were high-grade dysplasia. (Table 2) Most of the lesions (103 eyes (60.3%)) were located at the limbal area involving both the cornea and the conjunctiva. Lesions in 50 eyes (29.2%) involved the conjunctiva only with mostly the bulbar part and in 18 eyes (10.5%) involved only the cornea. Among 153 eyes that the lesions located at the limbus and the conjunctiva part, the most common originated site was at a nasal quadrant in 70 eyes, followed by a temporal quadrant in 64 eyes, a superior quadrant in 5 eyes, and an inferior quadrant in 2 eyes, and 12 eyes had the lesion involved nearly entire limbus. The most common presenting clinical feature of the tumor was a papilliform mass in 79 eyes (46.2%), followed by leukoplakia lesion in 37 eyes (21.6%), gelatinous mass in 31 eyes (18.1%), combined form in 19 eyes (11.1%), and diffuse/ ulcerated mass in 5 eyes (2.9%). (Fig. 1) Among these, a lesion with hyperpigmentation was found in 15 eyes (8.8%). The tumor was associated with pterygium in 40 eyes (23.8%). In our study, we defined distant metastasis as tumors that spread to regional lymph nodes or other visceral organs, while tumors with locally invasive are tumors with intraocular or orbital invasion. At presentation, 9.4% of patients had intra-ocular or orbital invasion and 3.5% had distant metastasis. Most distant metastasis cases were spreading to the regional lymph nodes and only one case had lung metastasis. (Table 2).

**Table 2:** Histo- and cytopathology and tumor characteristics at presentation

Tumor characteristics (*N* = 171 eyes)	Eye (N, %)
Histopathologic and cytologic findings	
- SCC	79 (46.2%)
- CIN	92 (53.8%)
• Mild dysplasia	21 (12.3%)
• Moderate dysplasia	14 (8.2%)
• Severe dysplasia/carcinoma in situ	43 (25.1%)
• NA	14 (8.2%)
Location	
- Cornea	18 (10.5%)
- Corneo-conjunctiva (limbus)	103 (60.3%)
- Conjunctiva	50 (29.2%)
Clinical characteristics	
- Papilliform mass	79 (46.2%)
- Leukoplakic lesion	37 (21.6%)
- Gelatinous mass	31 (18.1%)
- Combined form	19 (11.1%)
- Diffuse/ulcerated mass	5 (2.9%)
Extension of the lesions with limbal involvement	
- < 3 clock hours	71 (42.3%)
- 3–6 clock hours	66 (39.3%)
- > 6 clock hours	31 (18.4%)
- Association with pigmentation	15 (8.8%)
- Association with pterygium	40 (23.8%)
- Local spreading^a^	16 (9.4%)
- Distant metastasis^b^	6 (3.5%)

^a^Local spreading: intra-ocular or orbital invasion

^b^Distant metastasis: to lung, cervical lymph node, pre, and post-auricular lymph node

### Treatments and outcomes

Thirty-five eyes were excluded due to fewer than 3 months of follow-up time after treatments. Thus, 136 eyes with a mean ± SD follow-up time after treatment of 20.8 ± 2.2 months (range 3–110), were analyzed for the treatment outcomes. A summary of the initial treatments for OSSN and the outcomes after treatments were demonstrated in Table 3.

**Table 3:** Initial treatments of OSSN and the outcomes

Treatment options	*N* = 136 (eyes, %)	Resolved	Partial resolved	Recurrent	Not improved
Surgical excision	82(60.3%)	65/82(79.3%)	1/82(1.2%)	16/82(19.5%)	-
- *Wide excision with cryotherapy*	*65(47.8%)*	*51/65(78.5%)*	-	*14/65(21.5%)*	-
- *Superficial keratectomy with alcohol ablation*	*17(12.5%)*	*14/17(82.3%)*	*1/17(5.9%)*	*2/17(11.8%)*	-
Topical MMC 0.02%	42(30.9%)	30/42(71.4%)	10/42(23.8%)	2/42(4.8%)	
Other surgeries:					
- *Exenteration*	4(2.9%)	4/4(100%)	-	-	
- *Enucleation*	2(1.5%)	1/2(50%)	1/2(50%)	-	
Radiation	6(4.4%)	-	5/6(83.3%)	-	1/6(16.7%)

The main initial treatment was wide excision with adjunctive cryotherapy in 65 eyes (47.8%). Superficial keratectomy with alcohol ablation was performed in 12.5% of eyes that the tumor involved only the cornea. In the excision group, 29 eyes received topical MMC treatment (range from 1–16 cycles) for tumor debulking before surgical excision. One eye in the surgical group had a partially resolved mass which was eventually cured after seven cycles of topical MMC treatment.

Primary monotherapy with topical MMC was given in 42 eyes (30.9%) with a mean treatment cycle of 4.2 (range 1–18 cycles). Among these eyes, the lesions resolved completely in 30 eyes. Ten eyes in the MMC group responded well to topical treatment, however, the patients were lost to follow up during the treatment, thus the outcomes were reported as partially resolved.

Exenteration or enucleation was performed in 4.4% of eyes who had tumor spreading into intra-ocular and/or orbital tissues at presentation. Among these eyes, all were completely cured, except one eye that had residual tumor mass on the lower fornix post-enucleation. This eye, then received adjuvant local radiation and eventually the tumor was completely resolved. Among 88 eyes that underwent surgical treatment (including excision, exenteration, and enucleation), 29 eyes (32.9%) revealed a positive surgical margin from histopathologic sections.

**Fig. 2:**
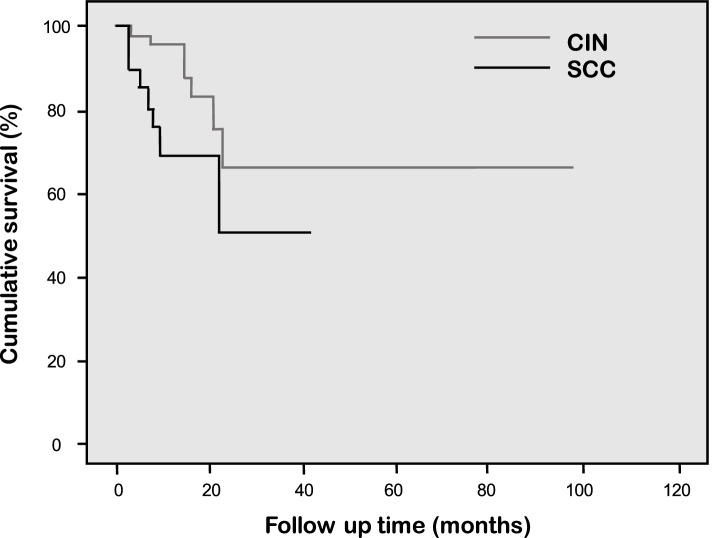
Recurrence Free Time Compared Between the Corneal-Conjunctival Intraepithelial Neoplasia and Squamous Cell Carcinoma

Recurrence of the tumor was found in 13.2% (18 eyes) which 11 eyes were SCC and 7 eyes were CIN. The mean ± SD time to recurrence was 15.0 ± 1.32 (range 2–48) months. Among 18 eyes with tumor recurrence, 11 eyes occurred within 1 year and 6 eyes had positive surgical margin. For the analysis of survival recurrence-free time, 17 eyes with partial resolved or not improved after primary treatment were excluded. The mean ± SD recurrence-free time for overall cases, SCC, and CIN subgroups was 65.9 ± 10.9 months (95% CI 44.5 – 87.3), 33.2 ± 4.6 (95%CI 24.0 – 42.3), and 81.3 ± 10.0 (95%CI 61.5 – 101.1), respectively. The mean recurrence-free time was significantly longer in the CIN than the SCC subgroup (*p* = 0.030). (Fig. 2) Considering all variables, SCC was the only significant risk factor that influences the recurrence of the tumors with the adjusted hazard ratio of 5.69 (*p* = 0.005) (Table 4).

**Table 4:** Factors influence the recurrence of the OSSN after treatments (*N* = 136 eyes)

Variable	Univariate analysis			Cox regression analysis		
	HR	95% CI	*P*-value	AHR	95% CI	*P*-value
Age	1.02	0.98–1.05	0.263	1.03	0.98–1.08	0.159
Sex	1.01	0.35–2.92	0.979			
Size of lesions < 3 clock hour	1.00					
3–6 clock hour	2.60	0.73–9.20	0.137		0.58–7.44	0.255
> 6 clock hours	3.61	0.96–13.50	0.056	2.09	0.32–6.05	0.651
Serology for HIV	1.29	0.64–2.61	0.463			
Pigmentation associated	0.04	0–182.82	0.462	1.40		
SCC	2.85	0.01–0.94	0.039	5.69	1.69–19.12	0.005*
Surgery	0.03	0–4.04	0.162	0.42	0.07–2.34	0.323

*Abbreviations*: *HR* Hazard ratio, *AHR* Adjusted hazard ratio

## Discussion

This study demonstrated that OSSN in Northern Thailand was more prevalent in males with an average age of 58 years. Similarly, previous studies reported that males were affected 2–4 times higher than females and most patients were more than 55 years old [18–25]. However, in a subgroup of seropositive HIV patients, the affected patients were of younger age (under 50 years). This finding was consistent with previous reports that OSSN in HIV-infected patients has been found to affect younger populations with a mean age at presentation in the third to fourth decade [26]. However, studies from Africa found either female predominates or no sex difference in HIV-positive OSSN patients [26].

This study found that the main presenting symptom was mass, followed by eye irritation, and visual impairment. Most of the lesions in this study were located at the interpalpebral area, particularly the limbal region. Previous studies also found that the most prevalent site of OSSN is located at the limbus [19, 23], indicating the location of heavy exposure to the ultraviolet (UV) light and the harbour site of stem cells which have unlimited regenerative capacities. In addition, nearly one-fourth of OSSN in this study were found in the same area of the pterygium site. Since both OSSN and pterygium are commonly reported with a higher incidence in tropical countries where people are chronically exposed to sunlight and UV radiation [2–4, 8, 27]. Also, sunlight exposure and UV irradiation are the most common risk factors for both the dysplastic change in OSSN and the development of pterygium. The reported concurrent rate of OSSN and pterygium varies from 5–29% [28–30]. Besides, HPV may be a viral co-factor in the pathogenesis of both pterygium and OSSN. From one review study, the prevalence of HPV in pterygium and OSSN was 18% and 33%, respectively. However, the role of HPV in pterygium and OSSN remains inconclusive due to the variable prevalence among studies (0–100%) [31].

The ulcerated mass or nodulo-ulcerative variant of OSSN is an uncommon characteristics according to the study of Kaliki et al. and is often misdiagnosed as necrotizing scleritis or sclerokeratitis [32]. In general, the macroscopic appearance of the OSSN has been described as one of three main types: leukoplakia, gelatinous, or papilliform; however, these appearances can be overlapped [6]. Moreover, some severe cases can have diffused mass with/ without ulceration, which is an uncommon variation. Palliform mass was the most common clinical finding of OSSN in our study, similar to other studies in an Asian population [21, 22]. However, studies from the Middle East and Indian populations reported that nodular gelatinous mass was the most common form [18, 19, 23, 24] and up to 10% of the lesions had hyperpigmentation [18, 24, 25]. This present study also revealed hyperpigmentation of mass in about 9% of eyes which may be related to racial skin pigmentation in the affected populations. Thus, OSSN can masquerade as pigmented tumor such as malignant melanoma or primary acquired melanosis [33]. Shields et al. reported a series of conjunctival CIN resembling melanoma occurred in both Caucasians and non-Caucasians. The authors postulated that the pigmentation may be due to intra-tumoral pigmented dendritic melanocytes [34]. It is important to exclude the melanocytic tumor from other lesions with pigmentation. In this study, the diagnosis in all cases was confirmed by the histopathologic or cytologic findings. In addition, these pigmented OSSN had typical characteristics suggesting the squamous cell in origin such as papilliform or hyper-keratinization, which also can be used as clues for diagnosis.

Since the diagnosis of OSSN cannot solely rely on clinical appearance, the gold standard for definite diagnosis remains tissue histopathology. Furthermore, the management of OSSN depends on the ability to distinguish between benign, pre-invasive, and invasive lesions. However, there are some drawbacks of performing tissue biopsy including the chance of tumor spreading, the scarring tissue from the repeated biopsy of suspicious lesions, the limbal stem cell deficiency, and patients’ discomfort. Impression cytology (IC) is an alternative method to diagnose the OSSN and to follow up after treatment. The method is a simple collection of the specimens from the ocular surface with numerous advantages including minimal discomfort, the ability to perform on an outpatient basis, and the precise location of the area being studied. Our previous study demonstrated that IC had high positive predictive accuracy (97.4%) in the diagnosis of OSSN compared with the gold standard tissue biopsy. However, the fair negative predictive accuracy (52.9%) of IC suggests that tissue biopsy remains necessary in cases of negative cytology [17]. Another study confirmed that cytologic exams can distinguish neoplastic from nonneoplastic lesions before tissue biopsy in as high as 80% of cases [35]. Moreover, IC is helpful as a non-invasive, diagnostic tool, particularly in a patient who is not a candidate for surgical treatment as well as during the follow-up period. Other less invasive methods for the diagnosis of OSSN include in vivo confocal microscopy (IVCM) and high-resolution spectral-domain optical coherence tomography (HR- OCT). IVCM is another way to assess morphologies of individual cells for dysplastic and neoplastic changes; however, the interpretation of the findings requires substantial technical expertise [36, 37]. High resolution (5–10 um) or ultra high resolution (3–5 um) spectral-domain OCT is useful in detecting epithelial thickness and differentiating epithelial from subepithelial lesions of the conjunctiva and cornea [38, 39]. HR-OCT, therefore, provides an “optical biopsy” and can assist in diagnosis, plan for surgical management, and follow up during treatments particularly in patients who receive topical treatment for OSSN [16, 40, 41]. However, these instruments are not available in every eye-care setting and require experienced and skilled technicians.

Even though OSSN is a low-grade malignancy, this study found that 9.4% of OSSN had local spreading to intraocular and orbital tissues. Previous studies reported that intraocular and orbital invasion was found in 2–13% and 12–16% of invasive cases, respectively [24, 42]. Distant metastasis of OSSN is also rare with a reported incidence of 0%-16% in cases of SCC and the first site of metastasis in regional lymph nodes [23, 42]. Although OSSN is a locally invasive tumor with a rare occurrence of distant metastasis, the patients with a delayed presentation to the healthcare system are at risk due to subsequent delays in diagnosis and treatments. In our study, 6 cases of metastasis were SCC with severe clinical manifestation at presentation and most of them had tumors spreading to regional lymph nodes. The diagnosis of SCC metastasis was confirmed by fine-needle aspiration biopsy of suspected nodes in all cases. All of these patients received external beam radiotherapy with unfavorable outcomes (Table 3).

Although surgery with wide excision and adjunctive cryotherapy is the traditional treatment for OSSN, conservative medical approaches have been increasingly used in recent years either in combination with surgical excision or as monotherapy [12, 15, 16]. In our setting, the decision for the treatments in each patient was determined on an individual basis including the clinical staging (size and area of involvement), the cytopathologic findings, and the presence of tumor invasion. Other associated factors that influenced the decision of treatment whether to perform surgery or conservative medical treatment included the patient’s baseline health status and compliance to local chemotherapy and follow-up after treatment. The new classification of OSSN proposed by Meel in 2018 also provided a rough guideline for the treatment, with a similar clinical approach to our practice [43]. Likewise, the issues of patient’s non-clinical characteristics including the possibility and co-operation for follow-up or continued treatment, economic and/or health status must be considered. Typically, the small tumors were treated by surgical removal, aiming for tumor-free margins (at least 4 mm), using the ‘‘no-touch’’ technique to avoid the potential risk of seeding comb. Cryotherapy was then applied to the conjunctival and limbal margins in a ‘‘double freeze slow thaw’’ technique, to achieve the ruptured membrane of residual tumor cells and the occlusion of the supplied blood vessels [44]. Adjunctive therapy whether cryotherapy or alcohol ablation should always be performed after the excision to prevent tumor local recurrence. Topical chemotherapy such as MMC was considered in the diffuse or large tumors either as monotherapy or in debulking the tumors before the surgery to minimize surgical complications. Patients with evidence of local spreading or metastasis received the operation of enucleation or exenteration and were also considered for external beam radiotherapy.

A survey of changes in the standard management of OSSN from 2003 to 2012, revealed a paradigm shift from surgical resection alone to an excision followed by adjuvant topical therapy. More than half of the physicians preferred using monotherapy with topical IFNα2b (56%) and MMC (37%) as the first-line treatment. IFN-α2b has replaced MMC as the agent most used for topical monotherapy, possibly because of a favorable safety and tolerance profile [12]. Because the toxicity profile of IFNα2b appears to be more favorable than that of MMC and 5-FU with similar rates of recurrence and complete response of the disease [13, 45]. One study, which compared between 5-FU and IFNα2b, found that both modalities resulted in a high frequency of tumor resolution and low recurrence rates and no difference in toxicity [46]. On the contrary, some studies found that MMC was the most effective treatment of both CIN and SCC despite its common short-term complications, which are often self-limiting [47, 48]. Though, IFN-α2b is a cost-effective treatment modality for OSSN in some countries [49], the cost of IFNα2b is still the main obstacle for usage in a developing country including Thailand. In this study, topical MMC 0.02% was used as the first-line treatment in 30% of cases and the results were satisfied in terms of the low rates of recurrence and complications, as well as lower cost compared to other agents such as IFNα2b.

Recurrence of OSSN after surgical excision has been reported in various rates from 10% to as high as 56% during a follow-up of 1 to 6.5 years. [1, 2, 10, 11, 19, 21, 22, 24, 50] This recurrence rate was significantly lower with adjuvant cryotherapy or postoperative topical medical therapy [21, 22, 51]. In the present study, the recurrence rate after surgical excision with adjunctive therapy was 11.8%. All surgical excision cases in this study did not receive postoperative topical MMC until the evidence of recurrence.

Previous studies reported that the risk factors of tumor recurrence after treatments included positive surgical margins [22, 23, 50, 51], postoperative use of topical chemotherapy [21, 22], tumor staging classified by tumor size and tumor local invasiveness, and higher pathologic grade [25, 48, 51]. In our study, patients with a positive margin after surgeries were closely monitored for recurrence that may need additional treatment. The reason for a high proportion of the positive margins may be partly due to the high proportions of cornea-conjunctival tumors which the free margin of the excised tissue from the cornea side was not likely. The positive surgical margin is accounted for 32.9% (29/88) for the surgery group. Among these, 18.2% (16/82) had tumor recurrence. This study showed that SCC was the only significant predictive factor for tumor recurrence. Notably, although the recurrence was higher in the surgery group compared with the topical MMC group, surgery was not a significant factor influencing the treatment outcome.

This study is probably one of the large cohorts of OSSN in South East Asia reporting the clinical characteristics and the treatment outcomes. However, there were some limitations to be addressed. Firstly, some data were missing due to the retrospective study design. Secondly, HIV and HPV blood screening tests were not done routinely in all patients. The roles of viral infection and OSSN warrants larger case–control studies. Lastly, the diagnosis of one-third of OSSN in this study was made by the impression cytology technique. This is partly because the preferred primary treatment of OSSN has been shifted from surgery to medical treatments.

In conclusion, OSSN in Northern Thailand primarily presents as a papilliform mass, particularly in the limbal area. HIV infection should be suspected in young patients. Managements were still surgical excision with adjunctive cryotherapy, even though, topical therapy with MMC also achieved satisfactory outcomes. Intra-epithelial neoplasia had better outcomes after treatment than invasive SCC. Besides some limitations, our data facilitates the understanding of OSSN in the Thai population. With reported clinical outcomes, our study support the new trends of assessment and treatment of OSSN which ultimately aim to reduce ocular morbidity and mortality associated with these ocular surface tumors.

## Data Availability

The datasets used and/or analyzed during the current study are available from the corresponding author upon reasonable request.

## Abbreviations

AHR: Adjusted hazard ratio
CIN: Corneal-conjunctival intraepithelial neoplasia
HIV: Human immunodeficiency virus
HPV: Human papillomavirus
HR-OCT: High-resolution spectral-domain optical coherence tomography
HR: Hazard ratio
IC: Impression cytology
IFN: Interferon-α2b
IVCM: In vivo confocal microscopy
MMC: Mitomycin C
OSSN: Ocular surface squamous neoplasia
SCC: Squamous cell carcinoma
UV: Ultraviolet radiation
5-FU: 5-Fluorouracil
